# Higher intracranial pressure variability is associated with lower cerebrovascular resistance in aneurysmal subarachnoid hemorrhage

**DOI:** 10.1007/s10877-022-00894-2

**Published:** 2022-07-17

**Authors:** Teodor Svedung Wettervik, Henrik Engquist, Timothy Howells, Anders Hånell, Elham Rostami, Elisabeth Ronne-Engström, Anders Lewén, Per Enblad

**Affiliations:** 1 Section of Neurosurgery, Department of Medical Sciences, Uppsala University, 751 85 Uppsala, Sweden; 2 Department of Surgical Sciences/Anesthesia and Intensive Care, Uppsala University, 751 85 Uppsala, Sweden

**Keywords:** Aneurysmal subarachnoid hemorrhage, Cerebral blood flow, Cerebrovascular resistance, Intracranial pressure variability, Xenon-enhanced computed tomography

## Abstract

Higher intracranial pressure variability (ICPV) has been associated with a more favorable cerebral energy metabolism, lower rate of delayed ischemic neurologic deficits, and more favorable outcome in aneurysmal subarachnoid hemorrhage (aSAH). We have hypothesized that higher ICPV partly reflects more compliant and active cerebral vessels. In this study, the aim was to further test this by investigating if higher ICPV was associated with lower cerebrovascular resistance (CVR) and higher cerebral blood flow (CBF) after aSAH. In this observational study, 147 aSAH patients were included, all of whom had been treated in the Neurointensive Care (NIC) Unit, Uppsala, Sweden, 2012–2020. They were required to have had ICP monitoring and at least one xenon-enhanced computed tomography (Xe-CT) scan to study cortical CBF within the first 2 weeks post-ictus. CVR was defined as the cerebral perfusion pressure in association with the Xe-CT scan divided by the concurrent CBF. ICPV was defined over three intervals: subminute (ICPV-1m), 30-min (ICPV-30m), and 4 h (ICPV-4h). The first 14 days were divided into early (days 1–3) and vasospasm phase (days 4–14). In the vasospasm phase, but not in the early phase, higher ICPV-4h (β =  − 0.19, p < 0.05) was independently associated with a lower CVR in a multiple linear regression analysis and with a higher global cortical CBF (r = 0.19, p < 0.05) in a univariate analysis. ICPV-1m and ICPV-30m were not associated with CVR or CBF in any phase. This study corroborates the hypothesis that higher ICPV, at least in the 4-h interval, is favorable and may reflect more compliant and possibly more active cerebral vessels.

**Supplementary Information:**

The online version contains supplementary material available at 10.1007/s10877-022-00894-2.

## Introduction

Intracranial pressure variability (ICPV) in acute brain injuries such as aneurysmal subarachnoid hemorrhage, aSAH [1–3] and traumatic brain injury, TBI [4–11] has received increased interest during the last several years. We have in recent studies found that higher ICPV is associated with a more favorable clinical outcome in both aSAH [2] and TBI [5]. In aSAH, we also found that higher ICPV correlates with a lower risk to develop delayed ischemic neurological deficits, DIND [2] and with better cerebral energy metabolism characterized by a lower rate of poor cerebral substrate supply [3]. Higher ICPV reflects a greater variation in cerebral blood volume (CBV) and is associated with unfavorable features such as higher blood pressure variability, which contribute to these CBV changes, and with a state of lower intracranial compliance, which amplify the effects of the CBV changes on ICP. However, considering the association between ICPV and favorable outcome, it likely also reflects beneficial features, such as cerebral vessels that are healthier, compliant and more active, possibly with less extensive atherosclerosis and cerebral vasospasm.

In this study, we aimed to improve the understanding of the ICPV concept, by investigating the actual relation between ICPV and global cerebrovascular resistance (CVR) and cerebral blood flow (CBF) in aSAH patients with ICP monitoring and CBF imaging during neurointensive care (NIC). Our hypothesis was that higher ICPV would be associated with a lower CVR and higher CBF.

## Materials and methods

### Patients

Patients with aSAH, admitted to the NIC Unit, at the Uppsala University Hospital, Sweden, between 2012 and 2020, were eligible for this study. All those 147 adult (age ≥ 18 years) patients who were intubated and mechanically ventilated with ICP monitoring and had at least one Xe-CT scan CBF imaging within the first 14 days after aSAH were included in the study.

### Treatment protocol

Patients were treated in accordance with our standardized ICP- and cerebral perfusion pressure (CPP)-oriented treatment protocol to avoid secondary insults, as described in detail in previous studies [12, 13].

### Data acquisition

ICP was monitored with an external ventricular drainage (EVD) system (HanniSet, Xtrans, Smith Medical GmbH, Glasbrunn, Germany or VentrEX, Neuromedex, Hamburg, Germany) and in a few cases with an extra intraparenchymal sensor device (Codman ICP Micro-Sensor, Codman & Shurtleff, Raynham, MA). Arterial blood pressure (ABP) was monitored in the radial artery at heart level. CPP was defined as the difference between mean arterial blood pressure (MAP) and ICP. Physiological data were collected at 100 Hz using the Odin software [14].

**Fig. 1:**
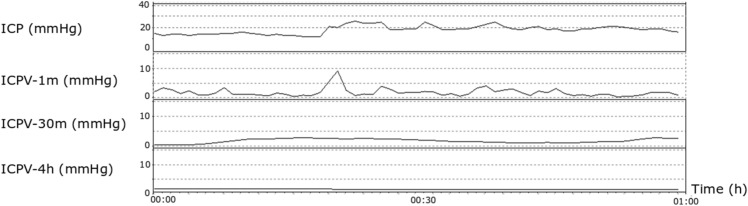
ICPV measures—example from one patient. The figure demonstrates the temporal course of ICPV in one patient over an hour of monitoring in association to a Xe-CT scan (time-point 00:30). ICPV-1m exhibited some temporal variation, whereas ICPV-4h was stable around 1 mmHg. *ICP* intracranial pressure, *ICPV* ICP variability, *Xe-CT* xenon-enhanced computed tomography

ICPV was analyzed in three ways with different time intervals (Fig. 1): (a) sub-minute (ICPV-1m), (2) 30-min (ICPV-30m), and (3) 4-h (ICPV-4h) [5, 15]. ICPV-1m was defined as the ICP slow wave amplitude with a bandpass filter, limiting the analysis to ICP oscillations with periods 55 to 15 s [15]. ICPV-30m and ICPV-4h were computed for every minute of monitoring as the absolute deviation from a 30-min and 4-h moving average centered on the minute, respectively [5]. The physiological variables were calculated from 15 min before to 15 min after the Xe-CT (30 min duration).

### Xenon-enhanced computed tomography and calculation of cerebral blood flow and oxygen delivery

**Fig. 2:**
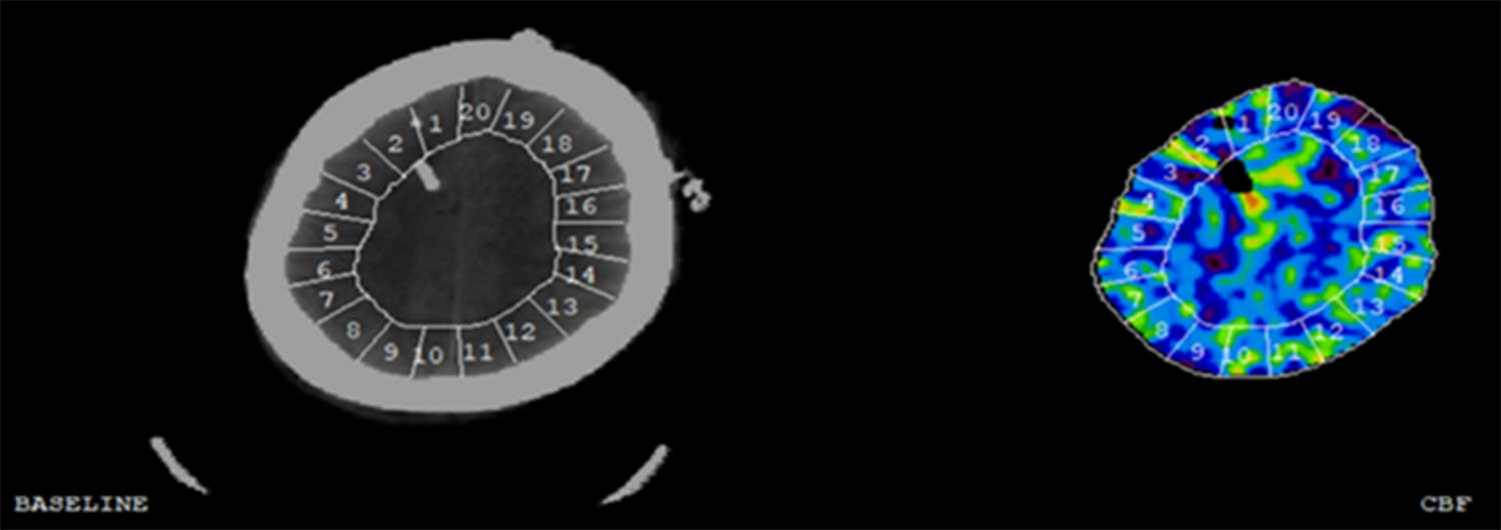
Cortical cerebral blood flow measurement—one example. The figure demonstrates cortical CBF in 20 different ROIs in a Xe-CT image. Global CBF was calculated based on average cortical CBF of typically three Xe-CT slices from the scan. Red areas indicate high CBF and blue areas low CBF. *CBF* cerebral blood flow, *ROI* region of interest, *Xe-CT* xenon-enhanced computed tomography

The Xe-CT CBF imaging procedures were done in accordance with the principles described by Gur et al. [16] and Yonas et al. [17, 18] and has been described in detail in previous studies by our group [19–23]. In brief, the procedure is based on the principle that inhaled xenon gas dissolves in blood and tissues. It may then act as a contrast agent for CT head scans which can be used for CBF calculations [24, 25]. The Xe-CT scans were conducted bedside in aSAH patients who were intubated and mechanically ventilated, in the NIC unit, using mobile CT. Non-radioactive 28% ^131^Xe was administered to the breathing circuit for 4.5 min, and CT scans synchronized to the xenon inhalation were obtained. Regional CBF in 20 cortical regions of interest (ROI; each around 350–450 mm^2^) of the CT image was calculated at four different levels of the brain (Fig. 2). The ROIs were visually inspected and single ROIs were occasionally removed in areas of hematomas or artefacts. Typically, three scan levels could be used for further calculations in each patient. Global cortical CBF in each individual patient was calculated as the mean value of all cortical ROIs (weighted by their ROI size). CVR was defined as CPP during the Xe-CT divided by global cortical CBF. The Xe-CT results were analyzed in the early phase (days 1–3) and vasospasm phase (days 4–14), separately.

### Outcome

Clinical outcome was assessed according to the Extended Glasgow Outcome Scale (GOS-E) 12 months after ictus, by trained personnel using structured telephone interviews. GOS-E ranges from death (1) to upper good recovery (8) [26, 27].

### Statistical analysis

The analysis primarily (i) aimed to determine the association of ICPV in relation to CVR and global cortical CBF.

The association between ICPV and CVR and cortical CBF was evaluated in the early phase (days 1–3) and the vasospasm phase (days 4–14) using the Spearman test. For patients with multiple Xe-CT measurements in each of the two phases, only the first scan of the phase was included. Multiple linear regression analyses were performed with CVR and global cortical CBF, respectively, as the dependent variable, and ICPV-4h in addition to age, WFNS grade, ICP, and CPP as baseline variables in the vasospasm phase. The regression analyses were limited to only ICPV-4h, not the other ICPV-measures, and only in the vasospasm phase, based on the significant findings in the univariate Spearman analysis. There were only a few missing observations and those were excluded from the analyses. A p-value < 0.05 was considered statistically significant. The statistical analyses were done using SPSS version 28 (IBM Corp, Armonk, NY, USA).

### Ethics

The study was approved by the Swedish Ethical Review Authority (Dnr 2020-05462). Written, informed consent was obtained during NIC from the next of kin.

## Results

### Patients, admission variables, treatments, and clinical outcome

In this study, 147 patients were included. Twenty-seven patients had done a Xe-CT only in the early phase, 73 only in the vasospasm phase, and 47 patients in both phases (first study analysed). The demographic, admission variables, treatments, and outcome variables are described in detail in Table 1.

**Table 1:** Demographics, admission status, treatments, and clinical outcome

Patients, n (%)	147 (100%)
Age (years), median (IQR)	61 (51–68)
Sex (male/female), n (%)	49/98 (33/67%)
WFNS grade, median (IQR)	4 (2–4)
Pupillary abnormality*, n (%)	3 (2%)
Fisher grade, median (IQR)	4 (3–4)
Aneurysm location (anterior/posterior), n (%)	124/23 (84/16%)
Aneurysm treatment (none/embolization/clipping/both), n (%)	2/121/23/1 (1/82/16/1%)
DIND, n (%)	51 (35%)
ICP monitor (EVD/EVD + Codman), n (%)	141/6 (96/4%)
Thiopental, n (%)	10 (7%)
Decompressive craniectomy, n (%)	15 (10%)
GOS-E*, median (IQR)	3 (3–5)
Favorable outcome**, n (%)	35/104 (25/75%)
Mortality**	23/116 (17/84%)

*DIND* delayed ischemic neurological deficit, *EVD* external ventricular drain, *GOS-E* Glasgow Outcome Scale-Extended, *ICP* intracranial pressure, *IQR* interquartile range, *SD* standard deviation, *WFNS* World Federation of Neurosurgical Societies. Italics indicate statistical significance

*Pupillary abnormality was defined as anisocoria and/or unreactive pupils

**8 Patients with missing outcome data

### Systemic and cerebral physiological variables during the cerebral blood flow imaging

The clinical and physiological data during the Xe-CT CBF imaging in the early phase (n = 74) and the vasospasm phase (n = 120) are described in Table 2.

**Table 2:** Systemic and cerebral physiological variables at the time point of xenon-enhanced computed tomography scans in the early phase and vasospasm phase

	Early phase (n = 74)	Vasospasm phase (n = 120)
ICPV-1m (mmHg), median (IQR)	1.9 (1.2–2.7)	1.6 (1.2–2.5)
ICPV-30m (mmHg), median (IQR)	1.9 (1.6–2.5)	2.1 (1.4–2.8)
ICPV-4h (mmHg), median (IQR)	2.3 (1.8–2.8)	2.2 (1.7–2.8)
ICP (mmHg), median (IQR)	15 (13–18)	14 (10–16)
CPP (mmHg), median (IQR)	75 (69–80)	78 (70–85)
CBF (mL/100 g/min), median (IQR)	33 (27–40)	35 (27–44)
CVR (mmHg/mL/100 g/min), median (IQR)	2.3 (1.8–3.0)	2.2 (1.8–3.0)

*CBF* cerebral blood flow, *CPP* cerebral perfusion pressure, *CVR* cerebrovascular resistance, *ICP* intracranial pressure, *ICPV* ICP variability

### Cerebrovascular resistance and cerebral blood flow in relation to intracranial pressure variability in the early phase and the vasospasm phase

**Fig. 3:**
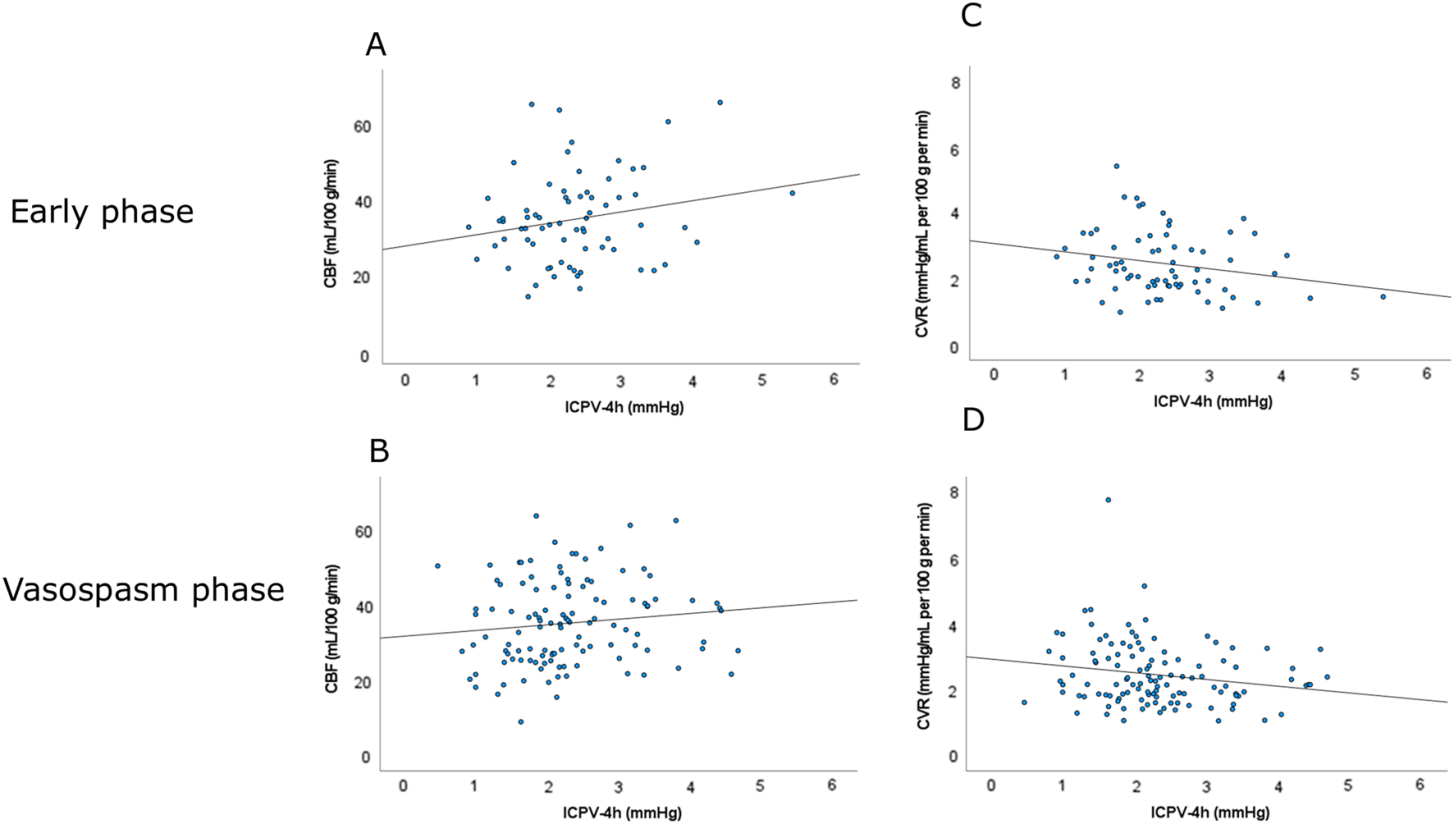
**A**–**D** ICPV-4h in relation to CBF and CVR in the early phase and the vasospasm phase. These scatter plots demonstrate the association between ICPV-4h and global cortical CBF in the early phase (**A**) and the vasospasm phase (**B**) as well as with CVR in the early phase (**C**) and in the vasospasm phase (**D**). Higher ICPV-4h was significantly associated with higher CBF (r = 0.19, p < 0.05) and lower CVR (r =  − 0.20, p < 0.05) in the vasospasm phase. *CBF* cerebral blood flow, *CVR* cerebrovascular resistance, *ICPV* intracranial pressure variability

Higher ICPV-4h was associated with lower CVR (r =  − 0.20, p < 0.05) and higher CBF (r = 0.19, p < 0.05) in the vasospasm phase (Table 3; Fig. 3), but only marginally with a lower CVR (r =  − 0.23, p = 0.053) and not with CBF (r = 0.15, p > 0.05) in the early phase. ICPV-1m and ICPV-30m were not associated with the CVR and CBF in any phase (Table 3; Supplementary Figs. 1, 2).

**Table 3:** ICPV in relation to CVR and CBF in the early phase and vasospasm phase—Spearman rank correlation analysis

Variables	Early phase			Vasospasm phase		
	ICPV-1m	ICPV-30m	ICPV-4h	ICPV-1m	ICPV-30m	ICPV-4h
CBF	− 0.19	− 0.02	0.15	0.00	0.07	*0.19*^a^
CVR	0.07	− 0.01	− 0.23	− 0.02	− 0.11	* − 0.20*^a^

*CBF* cerebral blood flow, *CVR* cerebrovascular resistance, *ICPV* intracranial pressure variability. Italics indicate statistical significance

^a^p < 0.05. The table indicates the r-values of the Spearman correlation analyses. Higher ICPV-4h was marginally associated with lower CVR (p = 0.053) in the early phase

In multiple linear regression analysis with CVR as dependent variable (Table 4), higher ICPV-4h was independently associated with lower CVR in the vasospasm phase (β =  − 0.19, p < 0.05) after adjustment for age, WFNS grade, Fisher scale, ICP, and CPP. In addition, higher CPP (β = 0.23, p < 0.05) was independently associated with higher CVR. In the multiple regression analysis with global cortical CBF as dependent variable, ICPV-4h (β = 0.14, p > 0.05) was not independently associated with global cortical CBF, after adjustment with the same independent variables as in the previously mentioned regression. However, higher WFNS grade was independently associated with lower global cortical CBF (β =  − 0.22, p < 0.05).

**Table 4:** ICPV-4h in relation to CVR and CBF in the vasospasm phase—a multiple linear regression analysis

Variables	(1) CVR (β)	(2) Global cortical CBF (β)
Age	0.07	− 0.14
WFNS grade	0.14	* − 0.21*^a^
Fisher scale	− 0.17	0.11
ICP	− 0.07	0.07
CPP	*0.23*^a^	0.02
ICPV-4h	* − 0.19*^a^	0.14

Italics indicate statistical significance

(1) R^2^ = 0.13, ANOVA, p = 0.02

(2) R^2^ = 0.08, ANOVA, p = 0.15

*CBF* cerebral blood flow, *CPP* cerebral perfusion pressure, *CVR* cerebrovascular resistance, *ICP* intracranial pressure, *ICPV* ICP variability, *WFNS* World Federation of Neurosurgical Societies

## Discussion

**Fig. 4:**
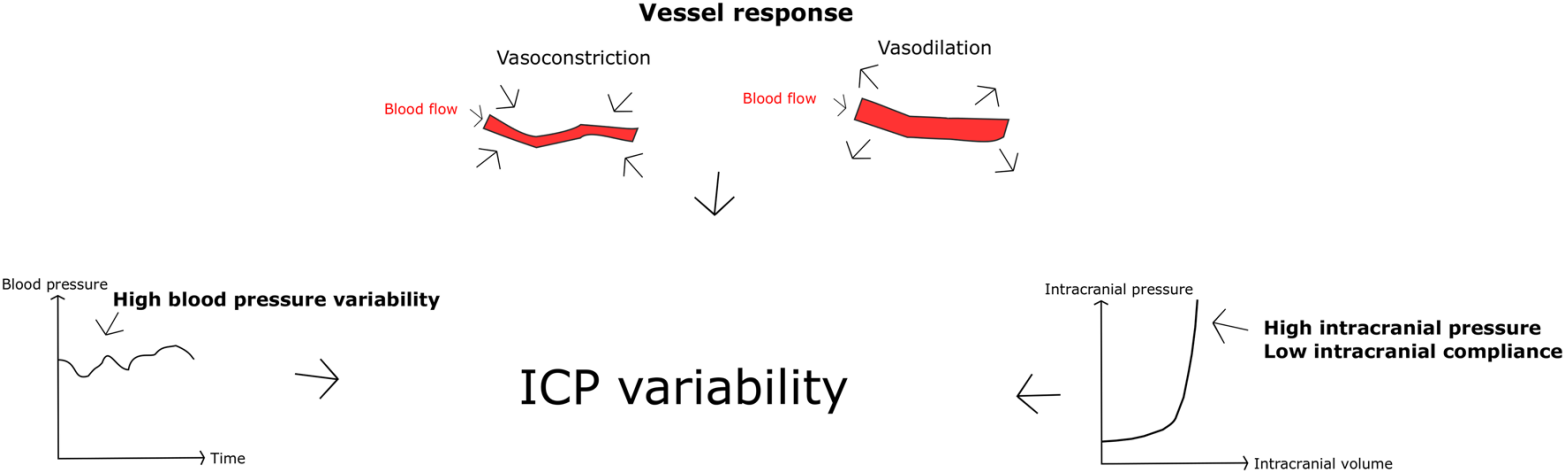
ICP variability—explanatory variables. We have previously demonstrated [5] that higher ICPV depends on variations in CBV as a consequence of higher BPV and likely also active and compliant cerebral vessels. The effect of the CBV variations on ICPV are amplified in a state of low intracranial compliance and high ICP. Hence, the suggested beneficial underlying mechanism of higher ICPV is supposed to be more active and compliant cerebral vessels predisposing for better CBF regulation and cerebral energy metabolic supply [3]

The interest in physiological variability has recently increased both in general [28, 29] and in acute brain injuries [1–10]. We have been particularly interested in the role of ICPV during the last years [2, 3, 5, 11]. First, we found that higher ICPV correlates with favorable outcome in TBI [5, 11], despite also being associated with unfavorable variables such as a low intracranial compliance and high blood pressure variability. In a large aSAH population, we found that higher ICPV correlates with favorable outcome in this disease as well and higher ICPV was also associated with a lower risk to develop DIND [2]. In a smaller study on aSAH with microdialysis monitoring, we then discovered that higher ICPV-1m was associated with a more favorable cerebral energy metabolic pattern, characterized by a lower rate of poor cerebral substrate supply [3]. Our hypothesis has been that high ICPV is a reflection of both unfavorable factors such as low intracranial compliance and higher blood pressure variability, but also favorable factors such as healthier, compliant and more active cerebral vessels. Specifically, more compliant and active vessels would be expected to generate greater changes in CBV and hence higher ICPV. These vessel characteristics would be expected to generate better CBF regulation with more optimal cerebral delivery of nutrients [3], as explained in Fig. 4. The favorable factors seem to predominate since ICPV is both uni- and multivariately associated with favorable cerebral physiology and clinical outcome [2, 5].

In the current study on 147 aSAH patients with ICP monitoring and CBF imaging, we found in the vasospasm phase that higher ICPV-4h was univariately associated with higher global cortical CBF and lower CVR, and this association held true for CVR in a multiple regression analysis. These findings corroborate earlier studies from our group, i.e. that higher ICPV is generally favorable and may reflect better CBF regulation with more compliant and active cerebral vessels. ICPV-1m and ICPV-30m were not associated with CVR and CBF. We have no clear answers for these findings. One potential explanation could be that ICPV in the 4-h interval might better reflect the relatively persistent increase in CVR that is thought to occur following cerebral vasospasm in aSAH, and that ICPV in the sub-minute and 30-min intervals might reflect more brief changes in cerebrovascular reactivity.

The implications of our earlier findings and the results of this study are that ICPV is an interesting variable that seems to reflect important aspects of the intracranial physiology. The ICPV measures change within a quite small range of 0–5 mmHg and it may still be difficult to use bedside considering all the factors that contribute to any change in ICPV. For example, higher ICPV in cases chiefly explained by a low intracranial compliance is likely not beneficial and could be associated with lower CPP and disturbed autoregulation. Instead, most likely, these ICPV measures may be of value as a part in multimodality monitoring to determine the probability of development of certain cerebral states such as imminent cerebral vasospasm, ischemia, and the risk of brain infarctions. Considering the complexity of analyzing such data, ICPV might be best suited as one of many variables for an integrated interpretation by an artificial intelligence systems as an aid in clinical decision-making in future NIC units.

### Methodological considerations

The strengths of the study was a relatively large patient population with high-resolution physiological data in combination with Xe-CT CBF imaging. The results were also consistent with previous theories and findings. There are also some limitations. The reliability of ICP wave form analysis with an open EVD has been questioned, since it may alter the ICP signal to some extent [30]. However, the validity of ICP slow waves seems to be preserved with an open EVD, which makes variables such as ICPV-1m valid [31, 32]. ICPV-30m and ICPV-4h evaluates the deviation of absolute ICP in relation to the average ICP for a 30-min/4-h time window and these two could have been influenced by an open EVD to some extent. The associations between ICPV-4h and CBF and CVR were relatively weak, however this was to some extent expected considering the vast amount of variables involved in the regulation of the cerebral vessels and CBF [22].

## Conclusions

Higher ICPV-4h was associated with a lower CVR and higher global cortical CBF in the vasospasm phase. This was consistent with our hypothesis that higher ICPV may reflect a greater variation in CBV partly due to more compliant and active cerebral vessels.

## Supplementary Information

Below is the link to the electronic supplementary material.

Supplementary file1 (DOCX 273 kb)

Supplementary file2 (DOCX 277 kb)

## Data Availability

Not available.
