# Supplementary material for: Higher intracranial pressure variability is associated with lower cerebrovascular resistance in aneurysmal subarachnoid hemorrhage
Source: J Clin Monit Comput. 2022 Jul 17;37(1):319–26. doi: 10.1007/s10877-022-00894-2 (PMC9852113; doi:10.1007/s10877-022-00894-2)
Supplement: Supplementary file 1 — Supplementary file1 (DOCX 273 kb) [file 10877_2022_894_MOESM1_ESM.docx]

**Supplementary figure 1. ICPV-1m in relation to CBF and CVR in the early phase and the vasospasm phase**

**
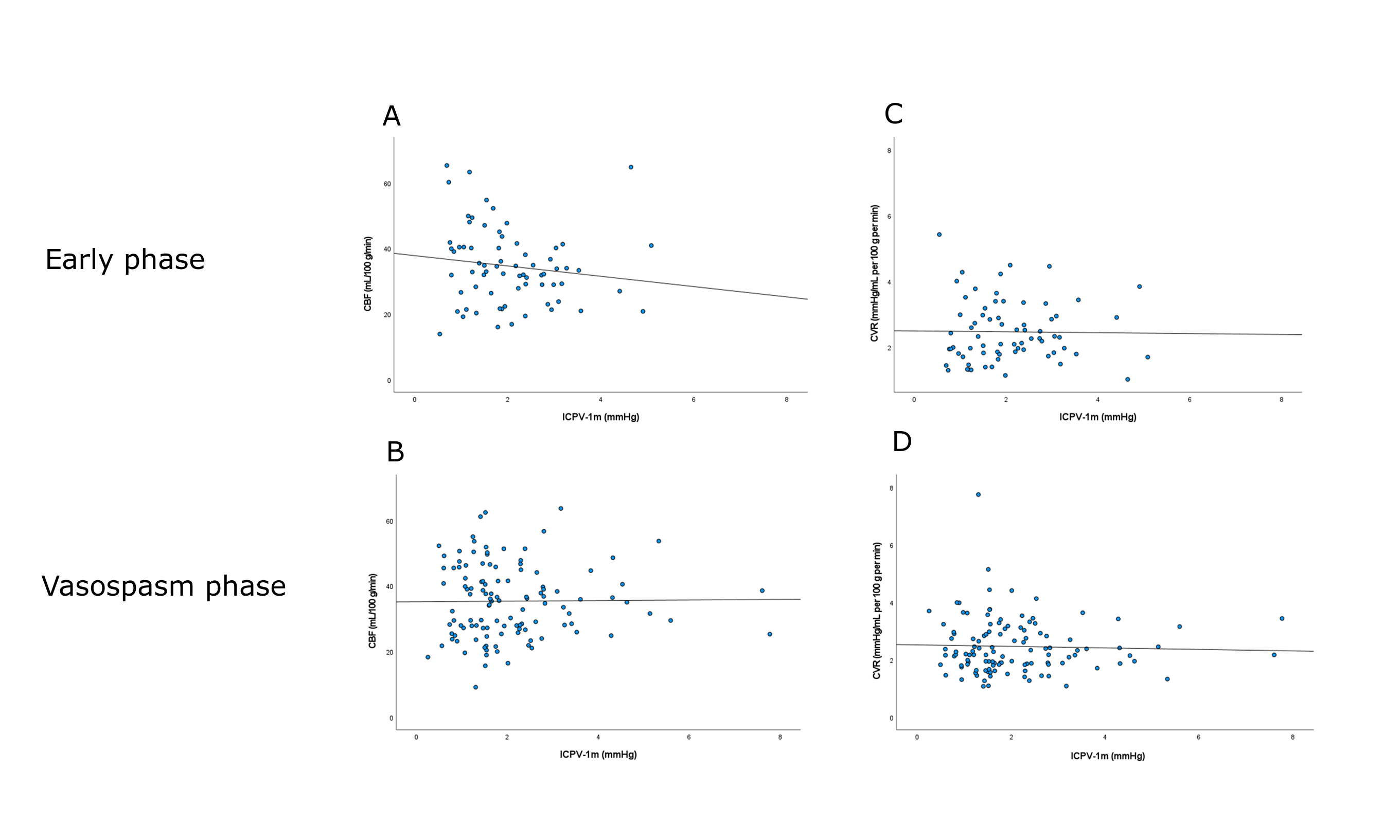
**

These scatter plots demonstrate the association between ICPV-1m and global cortical CBF in the early phase (1A) and the vasospasm phase (1B) as well as with CVR in the early phase (1C) and in the vasospasm phase (1D).

CBF = Cerebral blood flow. CVR = Cerebrovascular resistance. ICPV = Intracranial pressure variability.
